# Fibroblast-like cells accumulate late in human coronary atherosclerosis contributing to necrotic core formation

**DOI:** 10.1093/cvr/cvag002

**Published:** 2026-01-19

**Authors:** Daniel Morales-Cano, Diana Sharysh, Julián Albarrán-Juárez, Antonio de Molina, Verónica Labrador-Cantarero, Cecilie Markvard Møller, Laura Carramolino, Jacob F Bentzon

**Affiliations:** Centro Nacional de Investigaciones Cardiovasculares (CNIC), Calle Melchor Fernández Almagro, 3, Madrid 28029, Spain; Department of Clinical Medicine, Aarhus University, Palle Juul-Jensens Boulevard 11, 8200 Aarhus N, Denmark; Department of Clinical Medicine, Aarhus University, Palle Juul-Jensens Boulevard 11, 8200 Aarhus N, Denmark; Department of Clinical Medicine, Aarhus University, Palle Juul-Jensens Boulevard 11, 8200 Aarhus N, Denmark; Centro Nacional de Investigaciones Cardiovasculares (CNIC), Calle Melchor Fernández Almagro, 3, Madrid 28029, Spain; Centro Nacional de Investigaciones Cardiovasculares (CNIC), Calle Melchor Fernández Almagro, 3, Madrid 28029, Spain; Department of Clinical Medicine, Aarhus University, Palle Juul-Jensens Boulevard 11, 8200 Aarhus N, Denmark; Department of Cardiothoracic and Vascular Surgery, Aarhus University Hospital, Aarhus, Denmark; Centro Nacional de Investigaciones Cardiovasculares (CNIC), Calle Melchor Fernández Almagro, 3, Madrid 28029, Spain; Centro Nacional de Investigaciones Cardiovasculares (CNIC), Calle Melchor Fernández Almagro, 3, Madrid 28029, Spain; Department of Clinical Medicine, Aarhus University, Palle Juul-Jensens Boulevard 11, 8200 Aarhus N, Denmark; Steno Diabetes Center Aarhus and Department of Cardiology, Aarhus University Hospital, Aarhus, Denmark

**Keywords:** Atherosclerosis, Smooth muscle cells, Phenotypic modulation, Necrotic core

## Abstract

**Aims:**

Proliferation of arterial smooth muscle cells (SMCs) and their modulation to alternative mesenchymal phenotypes is central to atherosclerotic lesion growth. It has been studied extensively in mouse models, but a detailed analysis of when and where different mesenchymal cell types accumulate in human atherosclerosis is lacking. This study mapped mesenchymal cell populations during the progression of human coronary atherosclerosis and explored their associations with disease processes in human carotid plaques.

**Methods and results:**

Multiplex immunostaining protocols based on single-cell RNA sequencing-validated markers were established to detect SMCs, putatively SMC-derived mesenchymal cell subsets expressing osteoprotegerin or lumican, and macrophages in sections of left anterior descending arteries from forensic autopsies. The material comprised 44 arterial segments from 38 individuals, spanning normal intima, adaptive intimal thickening, pathological intimal thickening, and fibroatheroma. Parallel analysis of carotid endarterectomy samples allowed examination of mesenchymal cell involvement in fibrosis, calcification, and apoptosis. Validated machine learning-assisted cell classification was used to phenotype entire plaques at high microscopic resolution. The combined mesenchymal cell population constituted the majority of plaque cells at all plaque stages. Cells co-expressing contractile and mesenchymal cell markers were present in normal human coronary arteries, but mesenchymal cells lacking contractile protein expression became prominent only at the fibroatheroma stage, where fibroblast-like lumican-expressing cells localized preferentially around the necrotic core. The mesenchymal cell subtypes showed no preferential co-localization with areas of fibrosis or calcification; however, secreted osteoprotegerin was found bound to calcium deposits. Fibroblast-like, lumican-expressing cells accounted for 38–54% of all apoptotic cells for which a cell origin could be determined.

**Conclusion:**

Putative SMC-derived mesenchymal cells without contractile protein expression expand at the fibroatheroma stage of coronary atherosclerosis. Fibroblast-like cells localize around the necrotic core region and account for many apoptotic cells in plaques, suggesting a role in necrotic core development.

## Graphical Abstract

**Graphical Abstract:**
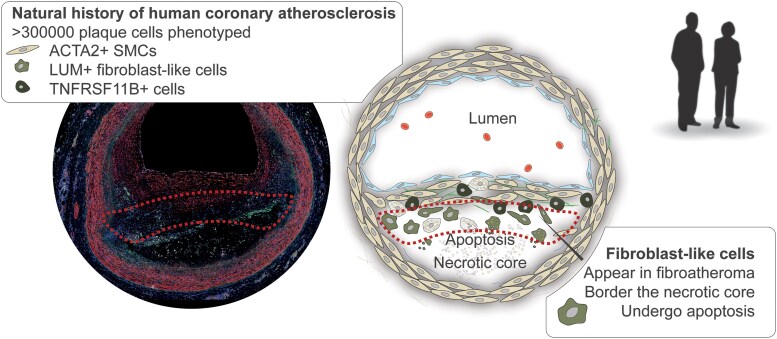

**Time of primary review: 45 days**

**See the editorial comment for this article ‘Architects of decay: how fibroblast-like mesenchymal cells shape the necrotic core’, by Y. Adachi**  ***et al*****., https://doi.org/10.1093/cvr/cvag025.**

## 1. Introduction

Smooth muscle cells (SMCs) reside in the walls of many tubular and hollow organs, including arteries, bronchi, intestines, and the bladder, and bestow upon these organs the ability to contract and dilate. Contractile function relies on contractile proteins, which also serve as markers of SMC identity. However, in diseases such as atherosclerosis, SMCs modulate to other mesenchymal cells with characteristics of fibroblasts and osteochondrogenic cells.^1–3^ These modulated SMCs, often referred to as fibromyocytes, chondromyocytes, or fibrochondrocytes,^1,4–6^ have been the subject of extensive lineage tracing studies in mouse models. Single-cell RNA sequencing (scRNA-seq) studies have identified similar cell types in human atherosclerosis, and analysis of SMC lineage-specific epigenetic modifications has, at least partly, confirmed their SMC origin.^7,8^ Endothelial cells undergoing endothelial-to-mesenchymal transition and invading adventitial cells constitute secondary sources of mesenchymal cells in mouse atherosclerosis, particularly under conditions where SMC recruitment is blocked or in uraemia-accelerated disease.^9–11^ Their quantitative contribution appears smaller in native murine atherosclerosis,^10,12^ and it remains unresolved for human atherosclerosis.

The transcriptional programs of plaque mesenchymal cells suggest an involvement in fibrosis and calcification, but little is known about their function. Moreover, it remains unknown at what stage of human atherosclerosis the different mesenchymal cell subtypes appear, where in the plaque they are located, or what spatial association they show with specific disease processes.

Such an analysis is not trivial. The first requirement is access to human plaque material that encompasses the full pathogenesis of atherosclerosis, including early lesion stages. These samples then need to be examined by multiplex immunostaining for several markers to distinguish different mesenchymal cell types from contractile SMCs and macrophages. Finally, determining the abundance and geographical localization of cell types in a plaque requires the phenotyping of thousands of cells at high microscopic resolution.

In the present study, we developed multiplex immunofluorescent staining protocols based on alpha-smooth muscle actin (ACTA2), the extracellular matrix proteoglycan lumican (LUM), and the secreted glycoprotein osteoprotegerin (TNFRSF11B) to identify subtypes of mesenchymal cells in autopsy samples of human coronary arteries, covering the range from normal intima to advanced fibroatheromas. We also implemented and validated an automated cell classification technique for accurate (low error rate) phenotyping of all cells in large human plaques. This analysis revealed that non-contractile mesenchymal cells, without ACTA2 expression, expand at the fibroatheroma stage, with fibroblast-like lumican-expressing cells localizing around the necrotic core. Analysis of freshly obtained plaques from carotid endarterectomies revealed that these cells account for a high proportion of apoptotic cells.

## 2. Methods

### 2.1 Human plaque material

Sections (5 µm) of proximal left anterior descending (LAD) coronary artery were obtained from a repository of arterial segments collected during forensic autopsies at the Institute of Forensic Medicine, University of Aarhus, Denmark, between 1996 and 1999.^13^ The samples were obtained from individuals aged 20–80 years, irrespective of the cause of death, with authorization from the Regional Research Ethics Committee (1996/3508) and the Danish Data Protection Agency, in accordance with the Declaration of Helsinki. Informed consent from next of kin was waived. The sample collection is anonymized and is not linked to any clinical information except for sex and age group (<45 or ≥45 years). Samples from individuals ≥45 years old were decalcified in 10% formic acid for 24 h before paraffin embedding.

Blocks were selected according to the lesion classification of Dalager et al.^13^ Atherosclerosis stage was confirmed by staining sections with haematoxylin-eosin, with reclassification according to the Virmani classification if needed.^14^ Arteries lacking evidence of disease (foam cells, lipid pools, necrosis, or calcifications) were classified as having normal intima or adaptative intimal thickening (AIT), depending on whether the intima was thinner or thicker than the underlying media. Lesions with lipid pools but no mature necrotic cores were classified as pathological intimal thickening (PIT), and those with a necrotic core as fibroatheromas. This study examined a total of 44 paraffin blocks from 38 individuals, with tissue classified by arterial histology as normal intima (*n* = 11; 6 male and 5 female), AIT (*n* = 11; 6 male and 5 female), PIT (*n* = 12; 8 male and 4 female), and fibroatheroma (*n* = 10; 7 male and 3 female).

For the analysis of associated disease processes (plaque fibrosis, calcification, and apoptosis), fresh carotid plaques were collected from endarterectomies conducted at the Department of Vascular Surgery, Aarhus University Hospital. These samples were fixed in formaldehyde for 24 h immediately upon collection and then embedded in paraffin, and 5-µm sections were cut from *n* = 12 plaque segments from 8 plaques from 8 females. As the samples were anonymized and collected during standard surgeries (not for this study), ethics board approval was not required (Danish Act no. 593 of 14 June 2011 on Research Ethics Review of Health Research Projects).

### 2.2 Single-cell RNA-Seq dataset analysis to identify modulated SMC markers

To define mesenchymal cell populations and their markers, we analysed the publicly deposited single-cell RNA sequencing data for human coronary plaques from Wirka et al. and for carotid endarterectomies from Pan et al., Alsaigh et al., and Sukhavasi et al.^1,2,6,15^ The datasets (GSE131778, GSE155512, GSE159677, and GSE260657) were downloaded as FASTQs and processed with CellRanger (v6.1.1) and the hg38 human reference genome (Ensembl 109) to reduce preprocessing differences. Data were then analysed in Seurat version 4.0.4 running in R version 4.2.2.^16^ The parameters for the initial filtering were as follows: Detected genes < 4000; 300 < UMIs < 30 000; and <10% mitochondrial, <60% ribosomal, and <1% haemoglobin genes expressed. Doublets were removed with DoubletFinder, assuming a 15% doublet rate.^17^ Datasets were normalized individually with SCTransform and integrated using the CCA Seurat method.^18^ Coronary and carotid plaque cells were analysed separately after additional data cleaning to retain only cells with the following features: detected genes 1100–4000 (coronary) or 900–4000 (carotids); 1300 < UMIs < 20 000. Principal component analysis included 40 principal components, and UMAP embedding was run with 22 (coronaries) or 18 (carotids) principal components, followed by clustering. The clusters were manually annotated based on the expression of known marker genes (*PTPRC*, *TYROBP*, *PECAM1*, *PI16*, and *ACTA2*), and mesenchymal cell types were selected and reclustered. Cells expressing *PTPRC* (encoding CD45) or *TYROBP* were defined as contaminants from other clusters and removed. A pseudotime trajectory was inferred for coronary plaque data using Slingshot (dynverse suite) with the root in the contractile SMC cluster.^19^ The expression levels of selected genes (*ACTA2*, *TNFRSF11B*, and *LUM*) were then analysed along the pseudotime trajectory branch towards the main fibroblasts population. To determine the significance of inferred pseudotemporal changes in the expression of these genes, we fitted a generalized additive model (using the ‘GAM’ R package) with pseudotime as an independent variable and locally estimated scatterplot smoothing (LOESS) of gene expression as a dependent variable. To test the similarity of mesenchymal-cluster diversity and cell type composition in the coronary and carotid datasets, we transferred cluster names for mesenchymal cells to the carotid dataset and projected the carotid plaque data onto the UMAP structure of the coronary plaque data using the Seurat functions TransferData and MapQuery, respectively.^18^

### 2.3 Multiplex immunostaining

Slides were dewaxed, rehydrated, and transferred to 0.05% Tween 20 citrate buffer (10 mM, pH 6.0). To retrieve antigens, slides were then heated for 3 min in a pressure cooker (LAD sections) or a microwave oven at 350 W for 5 min (endarterectomy sections). After two washes with phosphate-buffered saline (PBS), sections were permeabilized by incubation for 10 min at room temperature in PBS, 0.3% Triton X-100 and then blocked for 1 h at room temperature with 10% normal goat serum (Life Technologies, 16210-072) in PBS, 0.1% Tween 20. Blocked sections were incubated overnight at 4°C with combinations of primary antibodies (details in Supplementary material online, *Table S1*), followed by washes and incubation for 1 h at room temperature with matched fluorophore-conjugated secondary antibodies (details in Supplementary material online, *Table S2*). Nuclei were stained with DAPI (Sigma-Aldrich 1.24653), and slides were mounted in SlowFade Antifade mounting medium (Invitrogen S36937). Adjacent sections were incubated with isotype control antibodies at the same concentrations used for the corresponding primary antibody incubations.

### 2.4 Analysis of established cell type markers in LAD sections

Confocal images of CD45/CD68/ACTA2-stained fibroatheroma sections were acquired with a Nikon AR1 confocal microscope at 40× magnification using a Plan Fluor 40×/1.3 Oil DIC H N2 Oil objective. Z-stack images (z-step 1 μm) were acquired from each lesion in predefined regions, including plaque shoulders, cap (sides and centre), and core border (sides and centre). Each image was collapsed into a maximal intensity projection, and DAPI-stained nuclei were identified with a macro in Fiji (ImageJ distribution).^20^ Each nucleated cell profile was then manually characterized for expression of CD45, CD68, and ACTA2.

### 2.5 Analysis of modulated cell-type markers in whole plaque sections

LAD and carotid endarterectomy sections were stained with CD68/ACTA2/LUM or CD68/ACTA2/TNFRSF11B antibody combinations and scanned with an AxioScan Z1 slide scanner at 20× magnification using a Plan-Apochromat 20×/0.8 M27 objective. Fiji (ImageJ distribution) was used to adjust brightness and contrast and to remove autofluorescence with the AFID plugin developed by Baharlou et al.^20,21^ Identical microscope settings and image processing parameters were used to image adjacent isotype control-stained sections in the same session.

Counting of cell phenotypes (marker expression profiles) across whole plaque sections was done by a single observer by training a cell classifier tool in QuPath (v0.2.3 and 0.3.2).^22^ Briefly, the Random Trees machine-learning algorithm was trained on 300–500 human-classified cells in 10 shoulder regions in 5 coronary fibroatheromas (for coronary lesion analysis) and 20 randomly selected regions in 10 endarterectomy sections (for carotid lesion analysis). The trained classifiers for each marker were then applied sequentially to phenotype all plaque cells in the coronary or endarterectomy samples. The machine learning analysis was validated by comparing its results with manual cell counts by linear regression for 5 CD68/ACTA2/TNFRSF11B-stained and 6 CD68/ACTA2/LUM-stained coronary fibroatheromas (containing approximately 12 000 nucleated cell profiles in total).

Cell types were counted throughout the intima or plaque in all sections. In LAD fibroatheromas, cell types were also quantified in specific plaque regions of interest (ROIs), defined according to a previously described plaque subdivision.^23^ The operator manually delineated the lumen, plaque (excluding underlying media), and the necrotic core (the plaque region devoid of cells except for scattered nuclei) and selected the two points on the luminal surface at the junction between non-plaque and plaque tissue. These coordinates set the limits for automated delineation of the *border zone* (the 100-µm deep plaque region surrounding the necrotic core), *shoulder regions* (plaque tissue ≤750 µm from the luminal points at the plaque–non-plaque junction), and the *luminal region* (non-shoulder plaque tissue at a depth of ≤200 µm from the lumen surface). To ensure that plaque tissue was assigned to only one ROI, areas fulfilling more than one criterion were assigned according to the following order of precedence: border zone, shoulder, luminal region. Plaque tissue not included in any of these ROIs was defined as *other*. In this way, the sum of the ROIs and the necrotic core area equalled the total plaque area.

### 2.6 Analysis of apoptosis in endarterectomy samples

To identify and phenotype apoptotic cells, sections of endarterectomy samples were stained using TUNEL (terminal deoxynucleotidyl transferase-mediated dUTP nick end labelling) with the In Situ Cell Death Detection Kit, Fluorescein (11684795910, Sigma) in combination with CD68/ACTA2, CD68/LUM, or CD68/TNFRSF11B immunofluorescence. Sections were counterstained with DAPI and scanned in an epifluorescence Leica DM6B upright microscope using the tile-scan Navigator module with an HCL APO 10×/0.3 W dipping objective. To confirm specificity, all acquisition and postprocessing settings were tested on sections stained with isotype control antibodies. Fiji was used to adjust brightness and contrast and to reduce autofluorescence from marker channels by subtracting an autofluorescence image obtained in an empty channel. Labelled cells were then automatically counted using QuPath version 0.2.3, as described above.

### 2.7 Alizarin red and aniline blue staining

Endarterectomy sections were stained using standard protocols with Alizarin Red or Aniline Blue (as a component of Masson’s trichrome) to detect calcified and fibrous tissue, respectively. Masson's trichrome staining was performed on ACTA2/LUM/CD68-stained sections after AxioScan Z1 acquisition and removal of the coverslip. Because antigen retrieval in citrate buffer (pH 6) removes calcium deposits, Alizarin Red staining was performed on adjacent sections to those stained for ACTA2/CD68/TNFRSF11B. Brightfield images were digitalized with an AxioScan Z1 slide scanner at 20× magnification using a Plan-Apochromat 20×/0.8 M27 objective. ROIs covering Aniline Blue- and Alizarin Red-stained areas were defined with the colour deconvolution and colour threshold plugins, respectively, in Fiji and transferred to corresponding ACTA2/CD68/LUM and ACTA2/CD68/TNFRSF11B images. The proportions of cell types inside and outside ROIs were then determined by automated cell counting in QuPath version 0.2.3, as described above.

### 2.8 Statistics

Cell fractions of each staining profile within plaques or plaque ROIs were compared across categories (plaque region, arterial morphology, and associated disease processes) using the Kruskall–Wallis test followed by Dunn's post hoc test, comparing either all categories or using a specific category as the comparator, as specified in the figure legends. Agreement between automated and manual cell counts was tested by linear regression. Calculations were performed in Prism 9 (GraphPad Software), and differences were considered statistically significant at *P* < 0.05. Statistical analysis for single-cell RNA-seq data is described above.

### 2.9 Code and protocol availability

The code used to analyse public scRNA-seq data, ImageJ macros, and a step-by-step protocol with an associated macro for automated cell-type phenotyping with Qupath version 0.3.2. can be downloaded from https://github.com/LAB-JFB.

## 3. Results

### 3.1 Abundance and localization of unaccounted mesenchymal cell populations in coronary fibroatheromas

**Figure 1:**
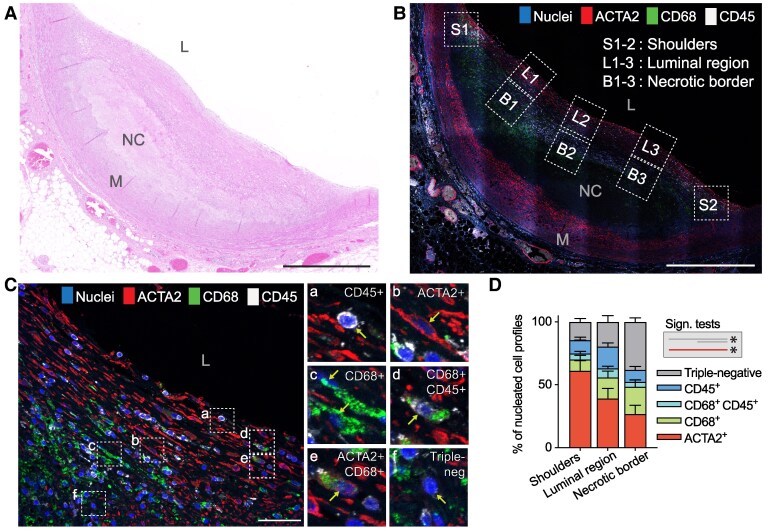
Rule-out approach with established cell markers to estimate the mesenchymal cell population in coronary fibroatheromas. (*A*) Representative haematoxylin–eosin-stained fibroatheroma from the human autopsy LAD plaque library. Scale bar, 1 mm. (*B*) The same plaque immunostained for ACTA2, CD68, and CD45. Scale bar, 1 mm. Boxes demark regions analysed at high magnification. (*C*) Example of a high-magnification view of a shoulder region (S2), showing examples of CD45+ hematopoietic cells (a), ACTA2+ SMCs (b), CD68+ cells (c), CD45+ CD68+ macrophages (d), rare ACTA2+ CD68+ cells (e), and cells negative for all three markers (f), identifying them as putative non-contractile mesenchymal cells. Scale bar, 50  μm. (*D*) Cellular composition in shoulders (S1–2), the luminal region (L1–3) and necrotic border zone (B1–3) (*n* = 9 independent fibroatheromas analysed with 1–2 segments per plaque). ACTA2+ cells predominate in the shoulder and luminal regions, whereas putative non-contractile mesenchymal cells (ACTA2/CD45/CD68-negative) are abundant in the necrotic core border. Bars indicate mean and SEM. Rare marker combinations that are not visible in the plot are not labelled. *P < 0.05 (comparisons indicated by line colour and length) by Kruskal–Wallis and Dunn's post hoc test. L, Lumen; M, Media; NC, necrotic core.

To map mesenchymal cells unaccounted for by traditional cell markers at different stages of coronary atherosclerosis, we examined a collection of LAD samples obtained from forensic autopsies of individuals aged 20–80 years, encompassing the full natural progression of lesion development.^13^ Coronary fibroatheromas (*n* = 9) were examined by multiplex immunostaining to identify contractile SMCs (ACTA2+), macrophages (CD68+), and lymphocytes and other hematopoietic cells (CD45+), with triple-negative cells being considered putative non-contractile types of mesenchymal cells (*Figure 1A–C*). In each section, we analysed eight regions per plaque at high magnification, including the two plaque shoulders, three areas in the luminal region, and three areas in the necrotic border region. ACTA2+ SMCs were mostly located in the luminal and shoulder regions, whereas triple-negative cells (putative non-contractile mesenchymal cells) constituted almost half of the cells near the necrotic core (*Figure 1D*). We also detected a large fraction of CD68+ cells not expressing CD45+. These could be CD68+ mesenchymal cells or macrophages with no detectable CD45 expression.

### 3.2 Markers of non-contractile mesenchymal cell types identified from scRNA-Seq data

**Figure 2:**
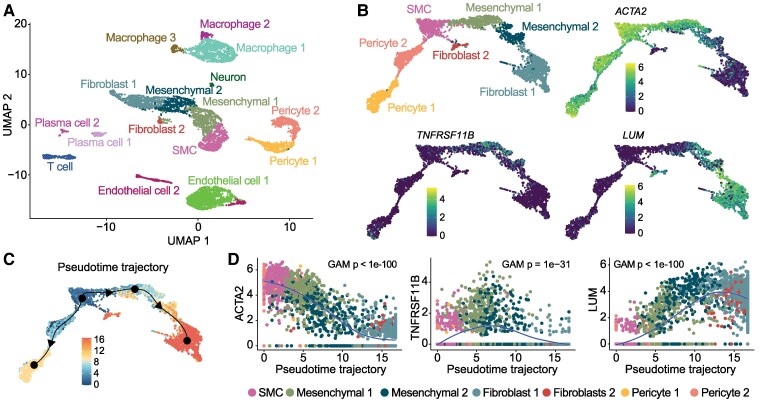
Marker analysis of publicly deposited single-cell RNA-Seq data from coronary artery plaques. (*A*) Clustering and cell-type annotation of the scRNA-Seq data from human atherosclerotic coronary arteries published by Wirka et al.^1^ (*B*) Visualization of marker gene expression in reclustered mesenchymal cell types (pericytes, SMCs, mesenchymal 1/2, and fibroblasts). Colour scale is normalized gene expression. (*C*) Inferred pseudotime trajectories from SMCs calculated with the Slingshot tool. Black dots denote ‘milestones’; arrows indicate the trajectory direction towards fibroblasts or pericytes. Colour scale is pseudotime coordinates. (*D*) Normalized expression of selected genes (ACTA2, TNFRSF11B, and LUM) ordered by inferred pseudotime trajectory (SMC-to-fibroblast 1 branch) and fitted with LOESS regression (blue curve). The association of gene expression with pseudotime coordinates was highly significant in a generalized additive model (GAM).

Previous scRNA-seq studies of advanced human coronary atherosclerosis reported lumican (*LUM*) and osteoprotegerin (*TNFRSF11B*) as marker genes of mesenchymal cells with fibroblast-like and osteochondrogenic-like phenotypes, respectively; however, they did not quantify the abundance and localization of these cell types during plaque development.^1,4^ To assess the performance of these markers for this type of analysis, we first re-analysed the coronary plaque scRNA-seq data from heart transplanted patients generated by Wirka et al.^1^ The mesenchymal cell supercluster, comprising contractile SMCs and other mesenchymal cells, was isolated and re-clustered, and the expression of *ACTA2*, *LUM*, and *TNFRSF11B* was analysed across the main axis of phenotypic diversity running from contractile, *ACTA2-*expressing SMCs to fibroblasts (*Figure 2A, B*). *TNFRSF11B* was expressed mainly by cells in the transition zone between *ACTA2*-expression and *ACTA2*-nonexpression, whereas *LUM* was expressed by fibroblast-like cells lacking *ACTA2* expression. To formally characterize these patterns, we used Slingshot to calculate a pseudotime trajectory running from contractile SMCs to fibroblasts, and then correlated pseudotime coordinates to the expression of *ACTA2*, *TNFRSF11B,* and *LUM* (*Figure 2C, D*). This analysis verified that *ACTA2*, *TNFRSF11B*, and *LUM* label sequential cell populations with phenotypes along the main axis of diversity from contractile SMCs towards fibroblasts. Similar results were obtained in a sub-analysis of the scRNA-seq data of only samples from patients transplanted for advanced coronary artery disease (see Supplementary material online, *Figure S1*). Together, *ACTA2*, *TNFRSF11B,* and *LUM* label most mesenchymal cells and are little expressed in non-mesenchymal cells (see Supplementary material online, *Figure S2*), making them good markers for mapping the entire population of mesenchymal cells in human atherosclerosis and roughly distinguishing their subtypes along the main axis of phenotypic diversity.

### 3.3 TNFRSF11B+ and LUM+ cells expand at the fibroatheroma stage of plaque development

**Figure 3:**
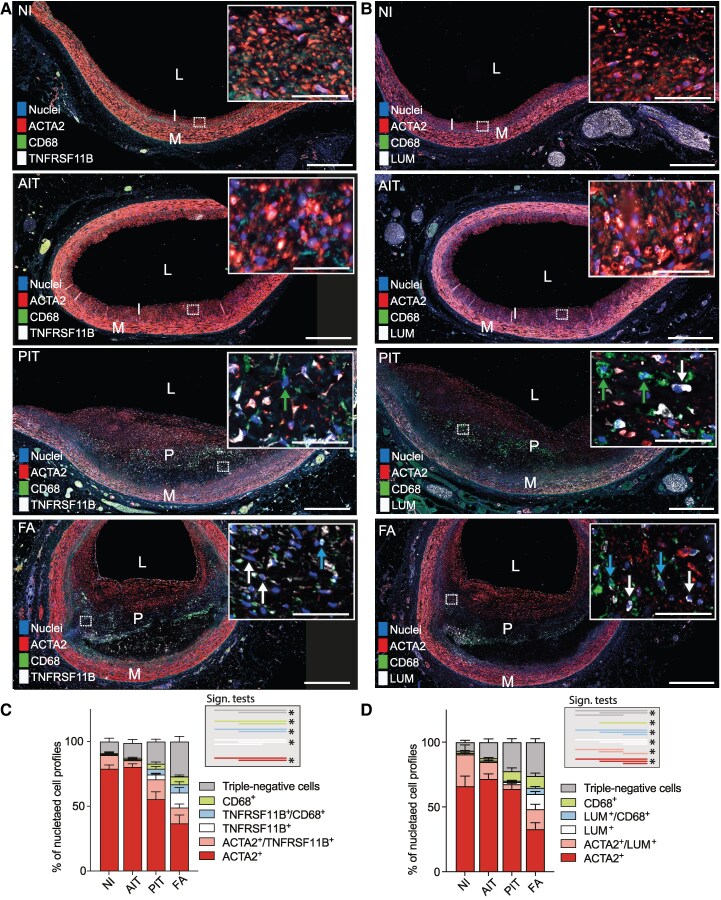
Non-contractile mesenchymal cells expand at the fibroatheroma stage. (*A, B*) Representative examples of ACTA2/CD68/TNFRSF11B and ACTA2/CD68/LUM staining in coronary artery sections with normal intima (NI), adaptive intimal thickening (AIT), pathological intimal thickening (PIT), and fibroatheroma (FA). Insets show cells at higher magnification. Arrows in PIT and FA panels point to examples of marker-expressing cells coloured according to the code in the graphs in C and D. Scale bars, 500 μm in overviews and 50 μm in insets. (*C, D*) Cell phenotype (marker expression profile) according to lesion stage, showing that fully modulated TNFRSF11B+ or LUM+ cells, which have lost expression of ACTA2, accumulate at the fibroatheroma stage. Bars show mean and SEM. Data points within each lesion category represent independent patients (NI, *n* = 8; AIT, *n* = 9; PIT, *n* = 12; FA, *n* = 10) with 1–3 sections analysed per patient. Rare marker combinations that are not visible in the plot are not labelled. *P < 0.05 for the indicated comparisons by Kruskall–Wallis test followed by Dunn's post hoc test. L, Lumen; M, Media; P, plaque; I, Intima.

To determine if cells expressing TNFRSF11B or LUM are present in normal intima or accumulate during atherosclerotic plaque formation, we established multiplex staining protocols for ACTA2/CD68/TNFRSF11B and ACTA2/CD68/LUM antibody combinations and stained sections of LAD with normal intima (NI, *n* = 8), adaptive intimal thickening (AIT, *n* = 9), pathological intimal thickening (PIT, *n* = 12), and fibroatheromas (FA, *n* = 10) (*Figure 3A, B*; isotype control staining in Supplementary material online, *Figure S3*). Multiple haematoxylin/eosin-stained examples of each lesion type are presented in Supplementary material online, *Figure S4*. Despite TNFRSF11B and LUM being secreted proteins, we found clear cellular staining of both markers. Indeed, the strongest signal with the anti-LUM antibody was within cells, suggesting that it binds better to the core protein, before GAG-chain modification. TNFRSF11B signal, with a dotted appearance, was also reproducibly observed in the necrotic core. To phenotype all plaque cells at single-cell resolution (a requirement for determining the cellular co-localization of markers), we then trained machine-learning-based tools in Qupath to automatically classify cell phenotype across the entire healthy intima or plaque. The tool was validated by comparison with approximately 12 000 manually curated cell types in fibroatheromas stained for ACTA2/CD68/TNFRSF11B (*n* = 5) or ACTA2/CD68/LUM (*n* = 6). Linear regression between machine-learning and manual determinations of cell fractions showed a high level of agreement for all analysed cell types (see Supplementary material online, *Figure S5*).

The results of automated cell phenotyping are shown in *Figure 3C, D*. During lesion pathogenesis, there was a decrease in the fraction of ACTA2+ SMCs (with or without concomitant expression of other markers), whereas the numbers of CD68+ macrophages increased. Cells lacking both these markers also increased in abundance, and the identity of these cells could be partly resolved with the LUM and TNFRSF11B markers. Double-positive cells, expressing ACTA2 plus TNFRSF11B or ACTA2 plus LUM, were present in normal intima and throughout lesion development, but non-contractile mesenchymal cells, expressing TNFRSF11B or LUM but not ACTA2, were restricted to lesions and were substantially more abundant at the fibroatheroma stage, accounting each for 12% of all plaque cells. Interestingly, the ACTA2-negative TNFRSF11B+ and LUM+ populations included cells that also expressed CD68. In the human plaque scRNA-seq data, *CD68* expression is scattered among non-contractile mesenchymal cells (see Supplementary material online, *Figure S6*), and the specificity of *TNFRSF11B* and *LUM* expression for the mesenchymal supercluster is higher than that of *CD68* expression for macrophages (see Supplementary material online, *Figure S7*). This suggests that TNFRSF11B + CD68+ and LUM + CD68+ cells are more likely to be mesenchymal cells than TNFRSF11B+ or LUM+ macrophages—a conclusion supported by the observation that many CD68+ cells in fibroatheromas lack CD45 (as noted above). The appearance of LUM+ cells at the fibroatheroma stage was confirmed for both males and females separately; however, the study was not statistically powered to explore possible sex-specific differences (see Supplementary material online, *Figure S8*). Notably, the entire mesenchymal cell population—staining positive for either ACTA2, TNFRSF11B, or LUM—accounted for the majority of cells in both PITs and fibroatheromas (*Figure 3C, D*).

### 3.4 LUM+ cells are enriched around the necrotic core

**Figure 4:**
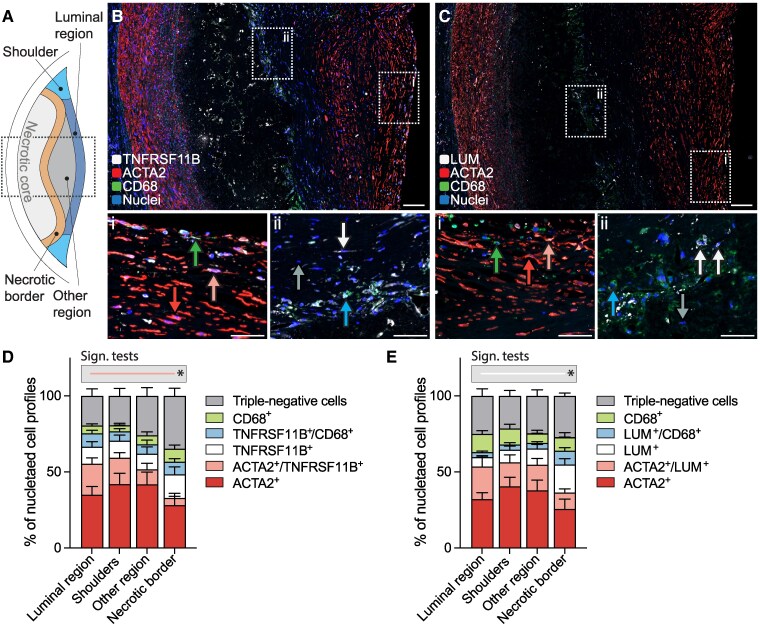
Fibroblast-like cells preferentially locate to the necrotic border region. (*A*) Schematic showing the fibroatheroma subdivisions in which cellular composition was analysed (see Methods for details). The boxed area corresponds to the sections shown in *B, C*. (*B, C*) Representative sections immunostained for ACTA2/CD68/TNFRSF11B and ACTA2/CD68/LUM, showing the differences in cellular composition along an axis from the lumen to the necrotic core. Arrows indicate examples of marker-expressing cells coloured according to the code in the graphs in *D* and *E*. Scale bars, 100 μm in overviews and 50 μm in high magnification images. (*D, E*) Cell phenotype across plaque regions, showing that LUM+ cells are significantly more abundant in the necrotic border region than in the luminal region (*n* = 10 fibroatheromas analysed). Bars show mean and SEM. Rare marker combinations that are not visible in the plot are not labelled. *P < 0.05 for the indicated comparison by Kruskall–Wallis and Dunn's post hoc tests.

To investigate the spatial distribution of the identified mesenchymal cell types in fibroatheromas, we segmented lesions into shoulders, luminal region, necrotic core border, and other regions (*Figure 4A*). Fibroblast-like LUM+ cells were significantly more abundant in the necrotic core border than in the luminal region of the plaque (*Figure 4B–E*). TNFRSF11B+ cells were similarly more abundant near the necrotic border, but in this case the difference did not reach statistical significance. Notably, the spatial association of LUM+ cells with the necrotic core is consistent with our observation that the accumulation of these cells coincides with necrotic core formation, when PIT lesions, lacking a necrotic core, transform into fibroatheromas.

### 3.5 Association of LUM+ and TNFRSF11B+ cells with fibrosis, calcification, and apoptosis

The extracellular-matrix proteoglycan LUM is involved in fibroblast-mediated deposition of fibrillar collagen,^24^ whereas the osteoblast-secreted soluble receptor glycoprotein TNFRSF11B (osteoprotegerin) protects bone from osteoclast resorption.^25^ These actions, coupled with the co-appearance and intraplaque location of cells expressing these markers near the necrotic core, suggested three possible implications in plaque development: an association of LUM+ cells with plaque fibrosis; an association of TNFRSF11B+ cells with plaque calcification; and an association of either of these markers with apoptosis.

We were unable to use the autopsy LAD samples to investigate these hypotheses because of extensive postmortem cell death and the decalcification of samples from individuals ≥45 years old. As an alternative, we obtained freshly processed carotid endarterectomies (examples of haematoxylin/eosin-stained sections in Supplementary material online, *Figure S9*). Analysis of publicly deposited scRNA-seq data for carotid endarterectomies confirmed that the mesenchymal cell phenotypes quantified in coronary atherosclerosis were also present in carotid plaques (see Supplementary material online, *Figure S10*). Multiplex analysis for ACTA2/CD68/LUM and ACTA2/CD68/TNFRSF11B on carotid plaque sections (*n* = 6) revealed that cells expressing only TNFRSF11B+ or only LUM+ accounted for 11 and 16% of all plaque cells, respectively (see Supplementary material online, *Figure S11*), similar to the 12% observed for each phenotype in coronary fibroatheromas.

**Figure 5:**
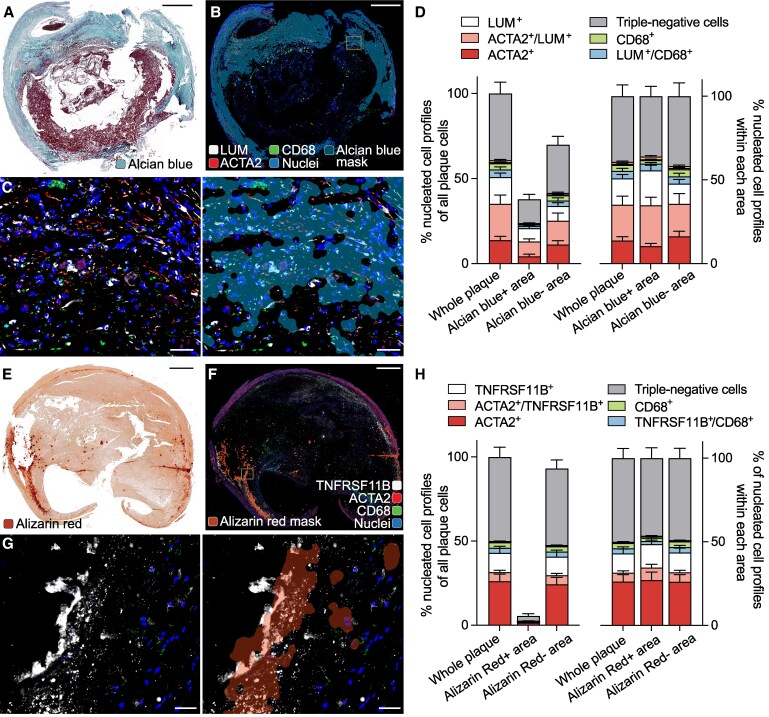
Association of mesenchymal cell types with fibrosis and calcification. (*A*) Representative staining of carotid plaque with Aniline Blue (as part of Masson's trichrome). (*B, C*) ACTA2/CD68/LUM staining on the same section (before Masson's trichrome), overlaid with a mask defined by high Aniline-blue signal in the Masson's trichrome staining. Panel *C* shows the boxed region at higher magnification with and without the mask. (*D*) Cellular composition inside and outside of the masked area, showing no differences according to fibrosity (*n* = 6 plaques from independent patients analysed with 1–2 segments per plaque). (*E*) Representative staining with Alizarin Red. (*F, G*) ACTA2/CD68/TNFRSF11B staining on an adjacent section, overlaid with a mask defined by Alizarin Red-detected calcium deposits. Panel *G* shows the boxed region at higher magnification with and without the mask. Note the positive staining of calcification granules. (*H*) Cell phenotype (marker expression profile) inside and outside of the masked area, showing few cells in the calcified (Alizarin Red+) region and no differences in cell composition (*n* = 6 plaques from independent patients analysed with 1–2 segments per plaque). All bars show mean and SEM. Rare marker combinations that are not visible in the plot are not labelled. Scale bars, 1000 μm (*A*, *B, E, F*) and 50 μm (*C*, *G*).

To investigate the co-localization of LUM+ cells with fibrotic connective tissue, we analysed ACTA2/CD68/LUM-stained sections by fluorescence microscopy and subsequently stained the same sections with Aniline Blue (as a component of Masson's trichrome) to identify collagen-rich tissue (*Figure 5A–C*). Regions rich in fibrous tissue harboured many LUM+ cells, but LUM+ cells were also found outside these regions, and no significant differences were detected between LUM+ cell distribution within and outside fibrotic regions (*Figure 5D*).

We used a similar approach to investigate the co-localization of TNFRSF11B+ cells with areas of calcification, this time staining with the ACTA2/CD68/TNFRSF11B antibody combination and Alizarin Red on adjacent sections (*Figure 5E–G*). TNFRSF11B and Alizarin Red staining partly overlapped; however, few cells were found near calcifications, and calcified areas were not enriched in TNFRSF11B+ cells (*Figure 5H*). Rather, the overlap in staining reflected the presence of TNFRSF11B on calcium deposits.

**Figure 6:**
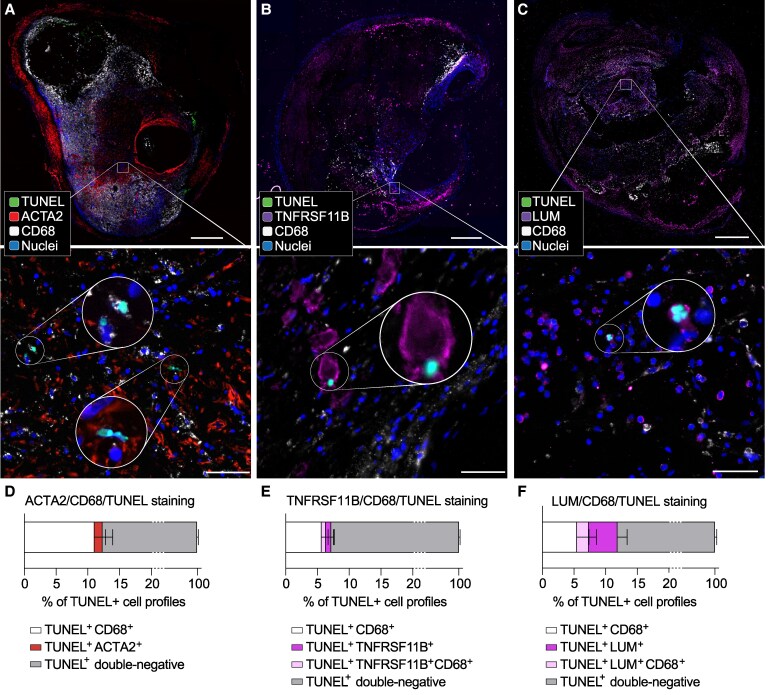
Contribution of SMCs and other mesenchymal cells to plaque apoptotic cells. *A–C*, Representative examples of TUNEL staining combined with immunofluorescence analysis for (*A*) CD68/ACTA2, (*B*) CD68/TNFRSF11B, and (*C*) CD68/LUM to determine the cellular origin of apoptotic cells. Boxed regions are shown at higher magnification in the lower panels, and circled insets show cells at still higher magnification. Scale bars, 500 μm in overviews and 50 μm in high magnification images. (*D–F*) Quantification of apoptotic cell phenotyping, showing that fibroblast-like LUM+ cells contribute substantially to apoptotic cells for which an origin can be inferred (*n* = 8 plaques from independent patients analysed, with 1–2 segments per plaque). No ACTA2+ CD68+ apoptotic cells were detected. Bars show mean and SEM.

To determine the phenotypes of apoptotic cells in plaques, we performed triple staining with TUNEL, CD68, and either ACTA2, TNFRSF11B, or LUM, to distinguish between apoptotic cells of macrophage, SMC, or other mesenchymal cell origin (*Figure 6*). Most TUNEL+ cells were negative for any marker, presumably due to marker-protein decay. Of the cell remnants for which an origin could be defined in TUNEL/CD68/ACTA2-stained sections, most were CD68+, whereas ACTA2+ apoptotic cells were uncommon. This is consistent with an earlier study that ascribed most cell death in plaques to macrophages based on this marker combination.^26^ However, antibody combinations including TNFRSF11B or LUM instead of ACTA2 identified contributions to apoptotic cells by the TNFRSF11B+ and LUM+ mesenchymal cells. This contribution was especially prominent in the case of LUM, with LUM+ cells contributing 38% and LUM+ CD68+ cells 16% of all apoptotic cells. Given that the scRNA-seq data identify LUM+ CD68+ cells as a subpopulation of LUM+ mesenchymal cells, this finding indicates that fibroblast-like LUM+ cells make a substantial and previously overlooked contribution to plaque apoptosis, accounting for as many apoptotic cells as macrophages in advanced human atherosclerosis.

## 4. Discussion

The proliferation and modulation of local SMCs to alternative mesenchymal cell types is a key process in the growth of atherosclerotic lesions. The temporal pattern of SMC-derived cell recruitment has been studied in mouse models,^27,28^ but analysis of human atherosclerosis has been restricted to advanced plaques obtained from hearts explanted during transplantation procedures or from carotid endarterectomies.^1,2,6,29^ There has thus been a lack of knowledge about when during atherosclerosis different mesenchymal cell phenotypes emerge. Furthermore, previous studies did not map the proximity of these cells to important plaque processes, such as fibrosis, calcification, and cell death, potentially useful information for defining their function in plaque development.

In the present study, we analysed the temporal and spatial accumulation of mesenchymal cell types during human coronary atherosclerosis using two previously reported markers, TNFRSF11B and LUM,^1^ which were further characterized in the present study for their ability to identify specific different mesenchymal cell populations. There is evidence that these mesenchymal cells in human atherosclerosis are at least in part SMC-derived,^7,8^ as is the case for the far majority of similar cell types in mouse atherosclerosis.^8,28^ However, some contribution from other sources, e.g. adventitial fibroblasts or endothelial cells transitioning to mesenchymal cells, cannot be excluded.

TNFRSF11B encodes the secreted glycoprotein osteoprotegerin, best known for its role in bone homeostasis.^25^ LUM encodes the extracellular matrix protein lumican, a small leucine-rich proteoglycan expressed by fibroblasts and previously implicated in myocardial fibrosis.^24^ Our re-analysis of the published scRNAseq data shows that LUM marks nearly all mesenchymal cells that have lost ACTA2 expression, whereas TNFRSF11B marks an intermediate phenotype. Both markers showed high specificity for distinguishing mesenchymal cells from other plaque cells, including macrophages. By establishing multiplex staining protocols and validated high-throughput image analysis techniques based on machine learning, we were able to phenotype the entire mesenchymal cell population in LAD and carotid plaques and link them to plaque stages and candidate plaque processes.

The multiplex immunofluorescence analysis showed that cells doubly positive for ACTA2 plus TNFRSF11B or for ACTA2 plus LUM are already present in normal arteries, whereas fully modulated TNFRSF11B+ and LUM+ cells lacking detectable ACTA2 expression are confined to atherosclerotic lesions and do not emerge until the fibroatheroma stage, coinciding with the appearance of the necrotic core. TNFRSF11B+ cells appeared to accumulate before LUM+ cells, consistent with a trajectory of modulation from contractile SMCs towards fibroblasts. That said, it remains unknown if SMC transformation to other mesenchymal cell phenotypes is gradual or instead involves rapid changes in phenotype, which would amount to cells jumping from one site to another in UMAP plots.

Consistent with their presence in fibroatheromas, the appearance of LUM+ cells coincided with necrotic core formation, and these cells were enriched in the zone bordering the necrotic core in coronary atherosclerosis. Furthermore, LUM+ cells contributed a substantial proportion of apoptotic cells in the carotid endarterectomy specimens. The contribution of SMCs to plaque cell death and necrosis has been a contested question. On the one hand, experimental studies have shown that forced apoptosis in plaque SMCs expands the necrotic core.^30^ On the other hand, human plaque studies phenotyping apoptotic cells for ACTA2 and CD68, as in the present study, have generally identified macrophages as the dominant source.^26^ As our analysis indicates, this may be because traditional SMC markers, such as ACTA2 do not detect the dying mesenchymal population. We found very few ACTA2+ apoptotic cells, whereas fibroblast-like LUM+ cells contributed on a par with macrophages, or even predominated if one considers LUM+ CD68+ cells as mesenchymal cells. The SMC origin of these dying cells is consistent with the transmission electron microscopy analysis of plaques by Kockx et al., which detected strangely shaped dying cells surrounded by basal laminae typical of SMCs.^31^ The abundance of LUM+ cells we detected near the necrotic core and their contribution to apoptotic cells raises the possibility that the trackway from contractile to fibroblast-like cells may be associated with a progressive increase in the susceptibility to apoptosis.

In murine atherosclerosis, cholesterol lowering depletes non-contractile mesenchymal cell types in the plaque interior.^12^ Assuming this effect is conserved in humans, current cholesterol-lowering therapies may thus stabilize plaques in part by reducing the non-contractile mesenchymal cells, which, as indicated by the present study, may help shrink the necrotic core. This therapeutic effect could be further enhanced by interventions that directly target the phenotypic transformation of SMCs, the proliferation of their modified progeny, or their death in the necrotic border zone. Supporting the feasibility of such strategies, multiple GWAS risk genes are expressed in mesenchymal cells,^32^ and a recent large cellular GWAS of apoptosis in cultured SMCs identified a locus that regulates SMC apoptosis and is nominally associated with the susceptibility to coronary artery disease.^33^

Our examination of the association between mesenchymal cells and other key pathogenetic mechanisms produced less conclusive results. Fibroblast-like LUM+ cells were present in fibrous plaque tissue but were not enriched at these locations compared with other regions. This might indicate that fibroblast-like cells with complete loss of ACTA2 expression are not required for the generation of fibrous tissue, which seems consistent with the wide expression of collagens by diverse mesenchymal cell types in human atherosclerotic plaque scRNA-seq data, including for ACTA2-expressing cells.^1,29^ Similarly, we found no evidence of physical proximity of TNFRSF11B+ cells to sites of calcification. However, secreted TNFRSF11B was localized in calcified regions, reminiscent of the detection of secreted TNFRSF11B on bone surfaces,^34^ where it reduces osteoclast activity by acting as a decoy receptor for the RANK ligand.^18^ In human and experimental atherosclerosis, calcification initiates in the necrotic core region,^35,36^ and unlike bone formation might not be tightly orchestrated by local cells. However, TNFRSF11B secreted by mesenchymal cells may help to stabilize expanding areas of calcification. Alternatively, the detected extracellular TNFRSF11B could be derived from mesenchymal cell-derived matrix vesicles, which have been implicated as nucleation sites of vascular calcification.^37,38^ The interpretation of previous studies in *Tnfrsf11b* knockout mice is complicated by excessive bone resorption, which may drive arterial calcification as a secondary effect.^39^ Further studies in mice with SMC-specific *Tnfrsf11b* deletion could be informative.

The study has several limitations. The coronary artery material was obtained from autopsies, and non-uniform postmortem protein degradation, previously documented by proteomics,^40^ may have influenced staining intensities. To address this limitation, freshly harvested carotid plaques were analysed to confirm the presence of the mesenchymal cell phenotypes identified in coronary atherosclerosis. Furthermore, the apoptotic cell analysis was performed on the carotid plaque material to avoid the distorting effect of postmortem apoptosis in the LAD autopsy samples. It should be noted that apoptosis represents only one mode of cell death in atherosclerosis, which happens to be detectable because apoptotic cells linger due to the suppression of efferocytosis.^41^ Other modes, such as necrosis, autophagic death, and pyroptosis, may also be important, but are difficult to detect because of their speed or lack of specific markers.^42^ Additionally, the relative rate of apoptosis for different cell types may be under- or over-estimated if cellular marker proteins are degraded at different rates or remnants cleared with different efficiencies.^43^

In conclusion, the present work demonstrates that specific types of mesenchymal cells, defined by expression of TNFRSF11B or LUM in the absence of ACTA2, emerge at the fibroatheroma stage of human coronary atherosclerosis. The fibroblast-like LUM-expressing cells are enriched around the necrotic core and account for a major fraction of apoptotic cells in human advanced plaques. Elucidating the mechanisms that drive the accumulation and death of these cells in the plaque interior may thus present a useful strategy for developing plaque-stabilizing therapies that can limit necrotic core formation.

Translational perspective Plaque mesenchymal cells—thought to derive mainly from expanding and modulating smooth muscle cells—are central to atherosclerosis progression and stability. Here, we provide the first map of their major subtypes during the natural history of human coronary atherosclerosis. We find that fibroblast-like cells are not present in normal arteries or early atherosclerosis but appear at the fibroatheroma stage, clustering around the necrotic core, and contributing to apoptotic cells on a par with macrophages. These findings may guide therapeutic strategies aimed at limiting necrotic core expansion and thereby reducing the incidence of plaque rupture and adverse cardiovascular events.

## Supplementary Material

cvag002_Supplementary_Data

## Data Availability

The scRNA-seq data used in this paper are accessible through the Gene Expression Omnibus with accession numbers GSE131778, GSE155512, GSE159677, and GSE260657. All other data are incorporated in the manuscript or supplementary material. Scanned images are available upon reasonable request.
