# Supplementary material for: Fibroblast-like cells accumulate late in human coronary atherosclerosis contributing to necrotic core formation
Source: Cardiovasc Res. 2026 Jan 19;122(3):336–48. doi: 10.1093/cvr/cvag002 (PMC13019684; doi:10.1093/cvr/cvag002)
Supplement: cvag002_Supplementary_Data [file cvag002_supplementary_data.zip › Morales_Supplement_CVR_R1.pdf]

Fibroblast-like cells accumulate late in human coronary atherosclerosis contributing to necrotic core formation

Daniel Morales-Cano, Diana Sharysh, Julián Albarrán-Juárez, Antonio de Molina, Verónica Labrador-Cantarero, Cecilie Markvard Møller, Laura Carramolino, Jacob F. Bentzon

Table of Contents

Supplementary Figure 1. Subgroup analysis of coronary plaque single-cell RNA-seq data from patients who underwent heart transplantation due to coronary artery disease.................................2

Supplementary Figure 2. Specificity of cell type markers..3

Supplementary Figure 3. Isotype staining controls. 4

Supplementary Figure 4. Histology of coronary artery samples 5

Supplementary Figure 5. Validation of trained automatic cell phenotyping 6

Supplementary Figure 6. Expression of CD68 along the axis of mesenchymal diversity. A, Clusters of mesenchymal cells 7

Supplementary Figure 7. Identity of cells co-expressing mesenchymal and macrophage markers.......8

Supplementary Figure 8. Cell composition in men and women during coronary atherogenesis 9

Supplementary Figure 9. Histology of carotid plaque samples. 10

Supplementary Figure 10. Markers of mesenchymal cells in carotid plaque scRNA-seq data............11

Supplementary Figure 11. Mesenchymal cell types in carotid plaques. 12

Supplementary Table 1. Primary antibodies used in the study. 13

Supplementary Table 2. Secondary antibodies used in the study..14

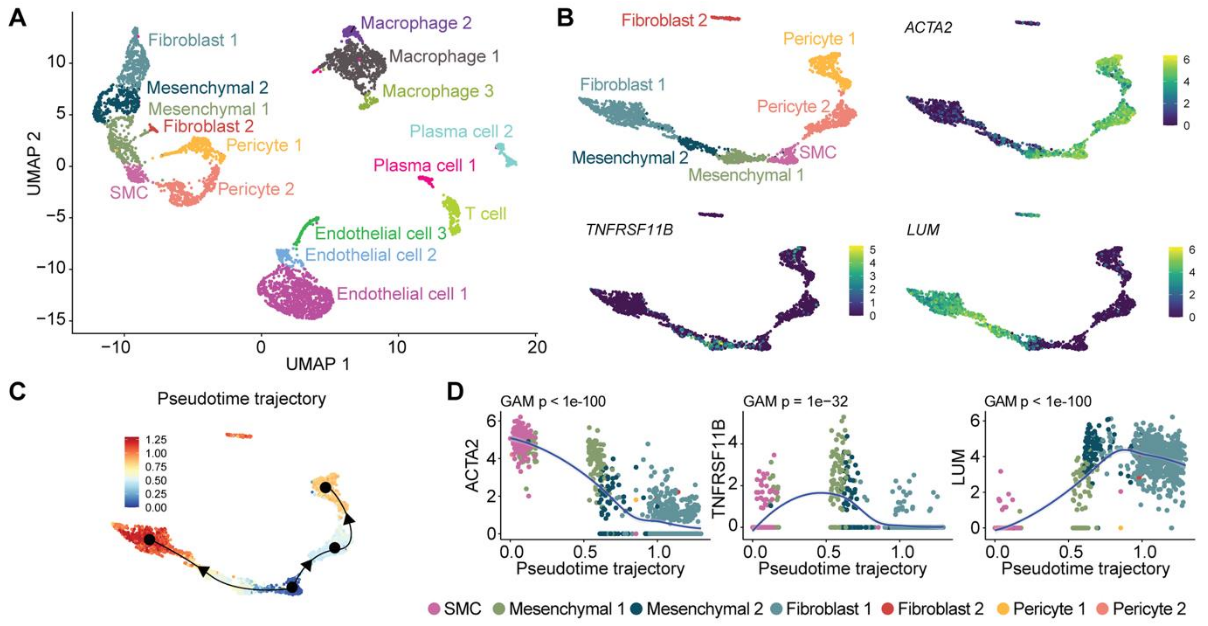

Supplementary Figure 1. Subgroup analysis of coronary plaque single-cell RNA-seq data from patients who underwent heart transplantation due to coronary artery disease. A, Clustering and cell-type annotation of the coronary plaque scRNA-seq data deposited by Wirka et al. (GSE131778). Only data obtained from the two patients transplanted for coronary artery disease are included. **B,** Visualization of marker gene expression in reclustered mesenchymal cell types (pericytes, SMCs, mesenchymal 1/2, and fibroblasts). Colour scale is normalized gene expression. **C,** Inferred pseudotime trajectories from SMCs calculated with the Slingshot tool. Black dots denote 'milestones'; arrows indicate the trajectory direction towards fibroblasts or pericytes. Colour scale is pseudotime coordinates. **D,** Normalized expression of selected genes (ACTA2, TNFRSF11B, and LUM) ordered by inferred pseudotime trajectory (SMC-to-fibroblast branch) and fitted with LOESS regression (blue curve). The association of gene expression with pseudotime coordinates was highly significant in a generalized additive model (GAM).

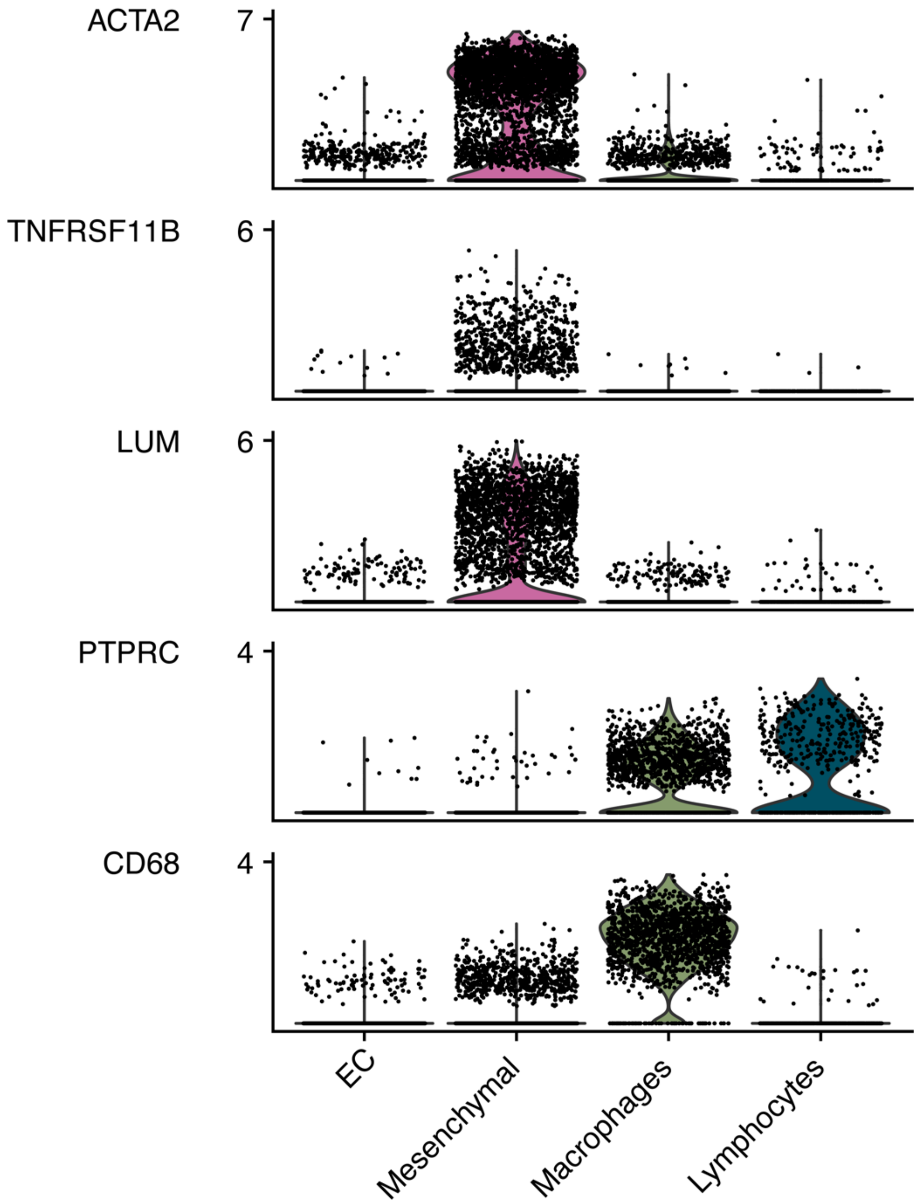

Supplementary Figure 2. Specificity of cell type markers. The violin plots show the normalized expression of the indicated marker genes in superclusters from the coronary atherosclerosis scRNA-seq data (GSE131778).

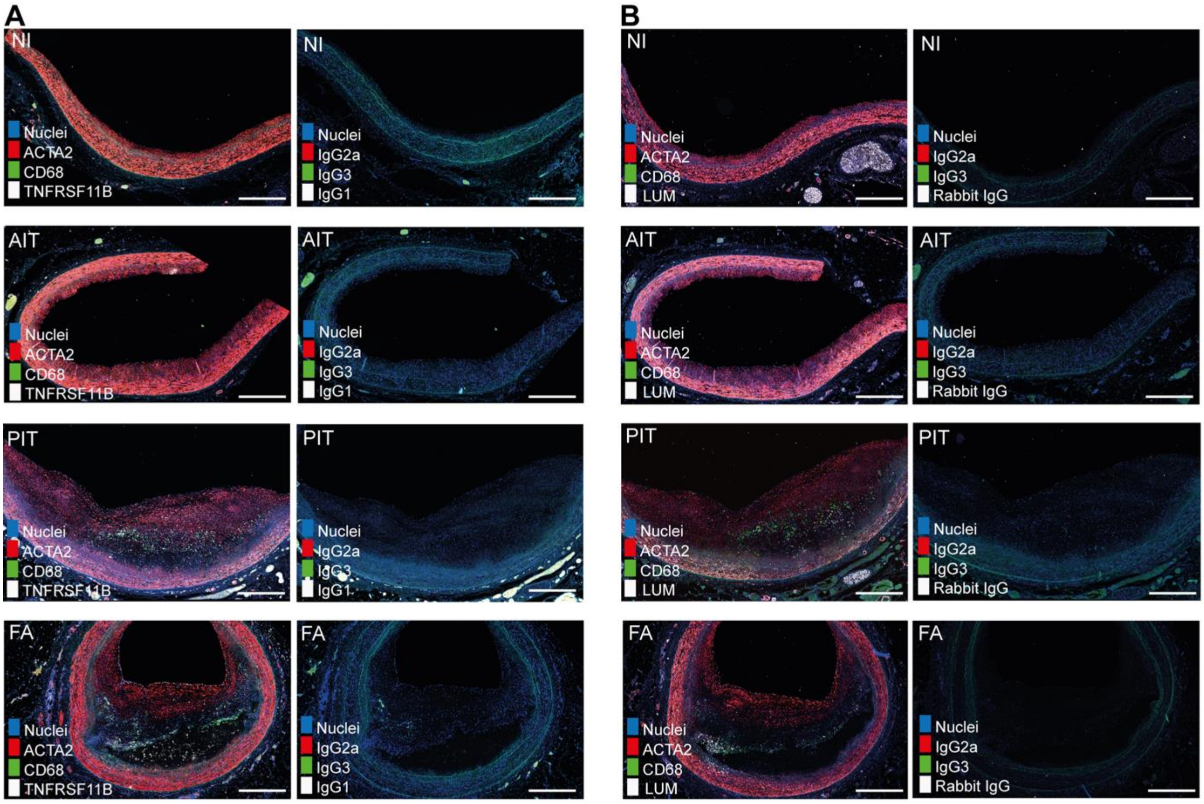

Supplementary Figure 3. Isotype staining controls. A, Panels show the images of ACTA2/CD68/TNFRSF11B-stained sections from Figure 3 alongside isotype staining controls performed on adjacent sections to confirm staining specificity. **B,** Similarly for ACTA2/CD68/LUM-stained sections and their isotype controls. Scale bars, 500 μm.

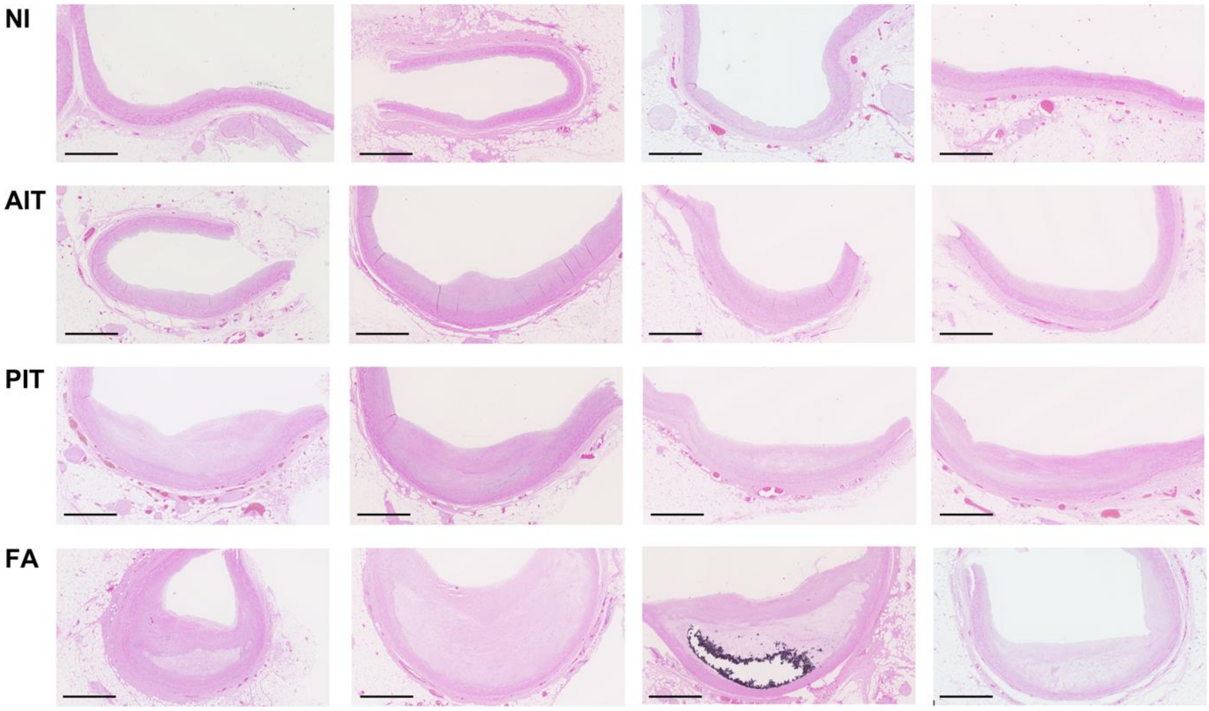

Supplementary Figure 4. Histology of coronary artery samples. Haematoxylin-eosin-stained sections of non-atherosclerotic left anterior descending arteries featuring normal intima (NI) or adaptive intimal thickening (AIT), and atherosclerotic arteries with pathological intimal thickening (PIT) or fibroatheroma (FA). Sections in the first column correspond to those stained in Figure 3. Scale bars, 500 μm.

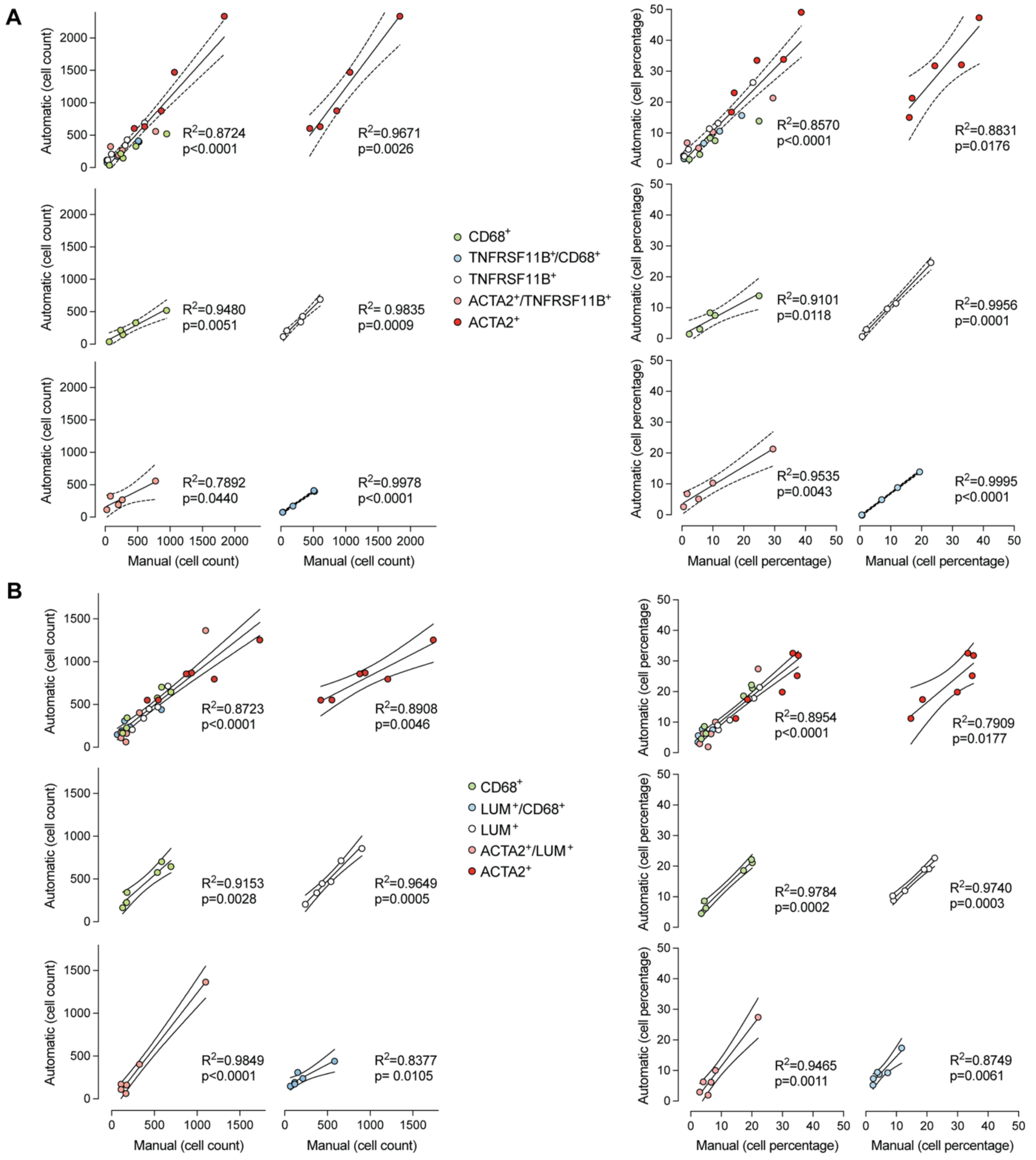

Supplementary Figure 5. Validation of trained automatic cell phenotyping. A-B, Comparison of automatic cell phenotyping in QuPath - based on the training of cell classifiers - with manually phenotyped cells, showing overall large agreement. Data represent cell counts (left) and percentages (right) in fibroatheroma sections stained for ACTA2/CD68/TNFRSF11B (A, n=5) and ACTA2/CD68/LUM (B, n=6). R^2 correlation coefficients and p values were calculated by linear regression.

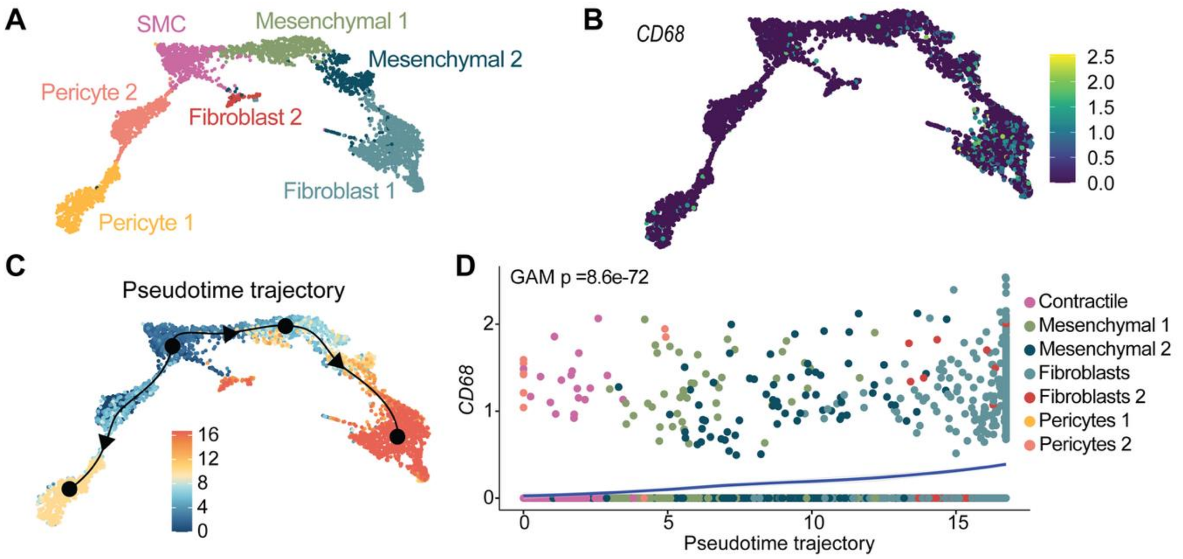

Supplementary Figure 6. Expression of CD68 along the axis of mesenchymal diversity. A, Clusters of mesenchymal cells in coronary plaque scRNA-seq data (GSE131778) (same as shown in Figure 2). **B,** Expression of *CD6*8. Colour scale is normalized gene expression. **C,** Inferred pseudotime trajectories from SMCs (same as shown in Figure 2). Colour scale is pseudotime coordinates. **D,** Normalized expression of *CD68* ordered by inferred pseudotime trajectory (SMC-to-fibroblast 1 branch) and fitted with LOESS regression (blue curve). The association of gene expression with pseudotime coordinates was highly significant in a generalized additive model (GAM).

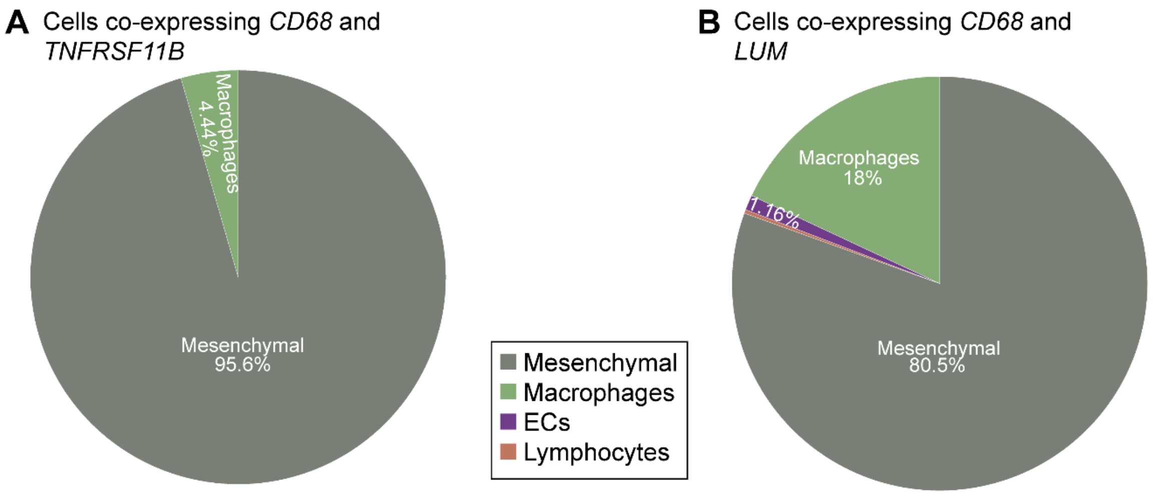

Supplementary Figure 7. Identity of cells co-expressing mesenchymal and macrophage markers. A-B, The likelihood of cellular identity was defined as the number of CD68/TNFRSF11B (A) or CD68/LUM (B) co-expressing cells in a cluster divided by the number of total co-expressing cells in coronary plaque scRNA-seq data (GSE131778).

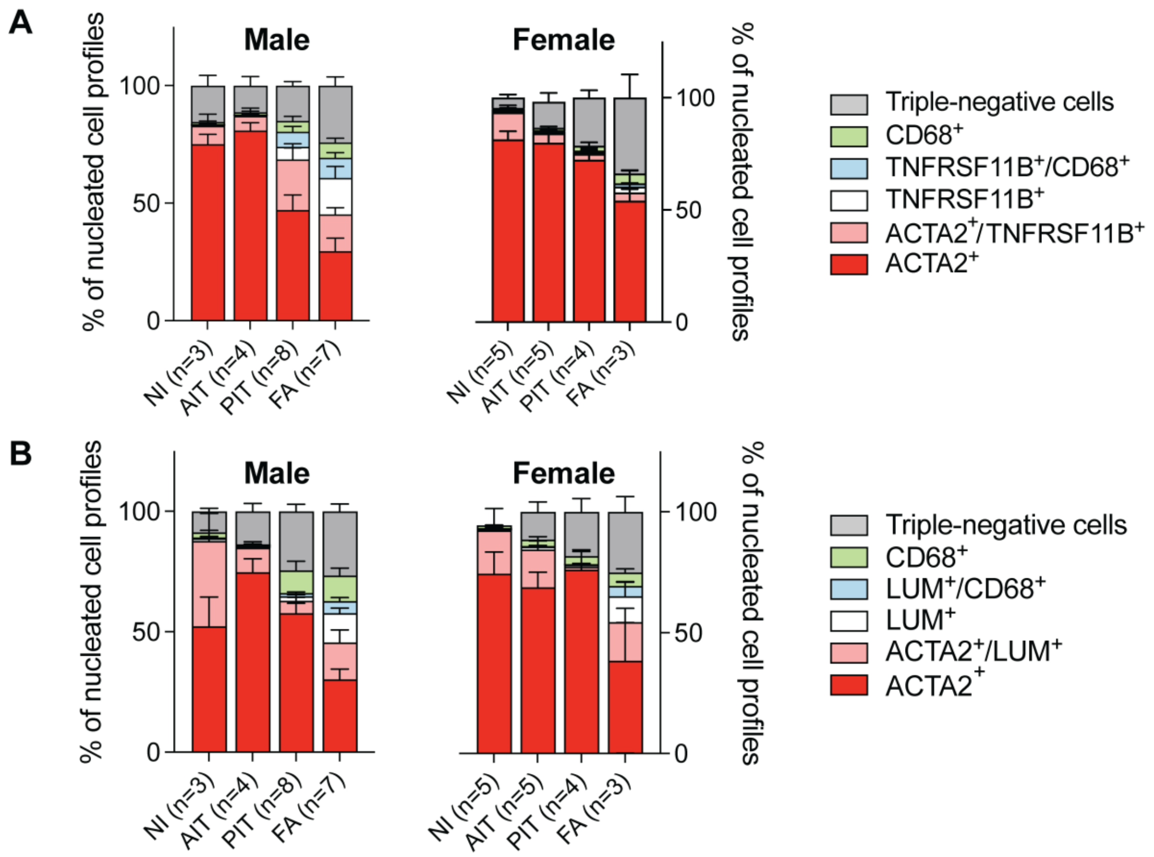

Supplementary Figure 8. Cell composition in men and women during coronary atherogenesis. A-B. Cell phenotype (marker expression profile) in sections stained for ACTA2/CD68/TNFRSF11B and for ACTA2/CD68/LUM in men and women. The plaque library included few advanced lesion stages from women, consistent with the slower atherosclerosis progression in women. Consequently, the pathological intimal thickening (PIT) and fibroatheroma (FA) categories contain fewer lesions from women. Nevertheless, the appearance of fully modulated LUM+ cells at the fibroatheroma stage is clear in both sexes. Bars show mean and SEM. NI, normal intima. AIT, adaptive intimal thickening.

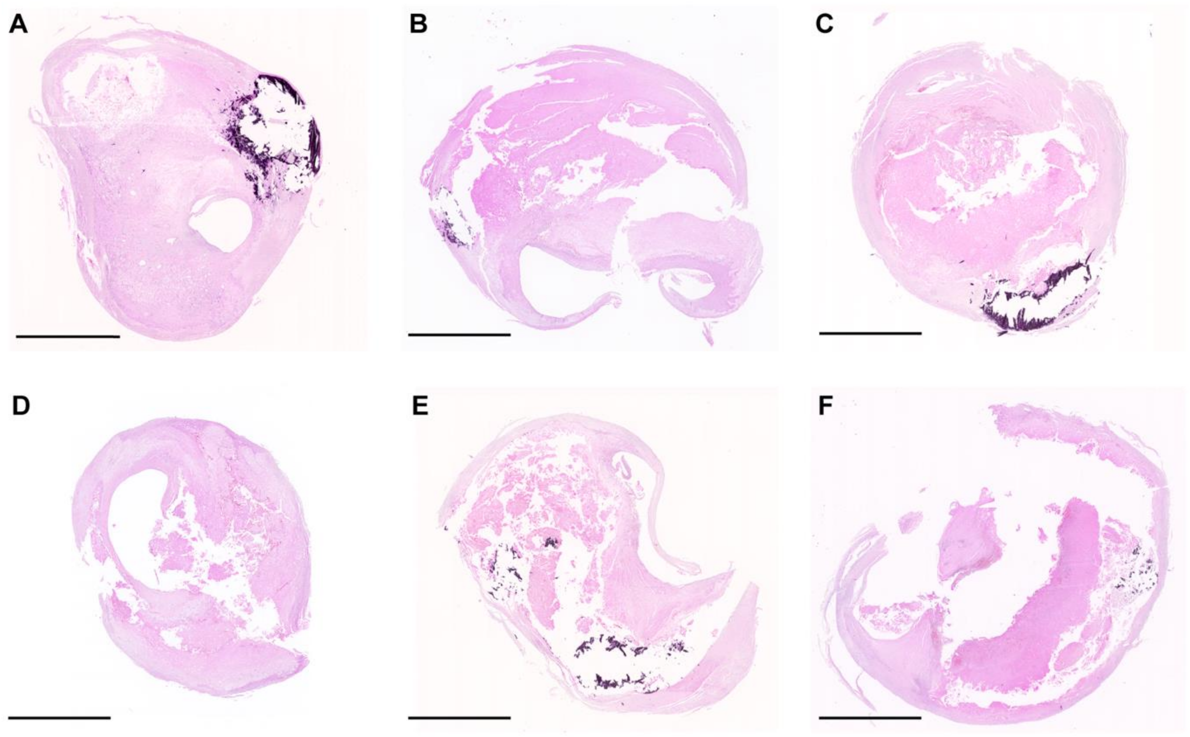

Supplementary Figure 9. Histology of carotid plaque samples. A-F, Examples of haematoxylin-eosin-stained sections of 6 carotid plaques obtained from independent patients, showing the advanced state of plaque development. The plaque in A correspond to that shown in Figure 6A, B to those shown in Figure 5E/6B, C to those shown in Figure 5A/6C, and D to that shown in Suppl. Fig. 11. Panel E and F are additional examples. Scale bars, 2.5 mm.

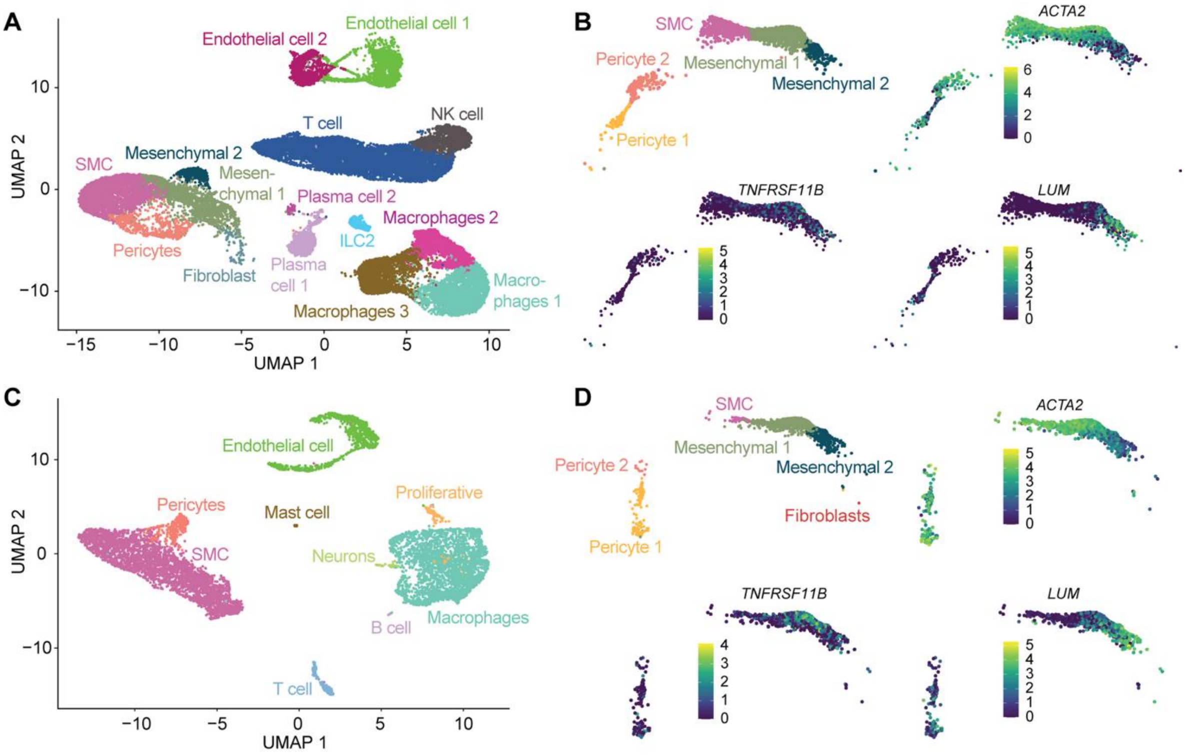

Supplementary Figure 10. Markers of mesenchymal cells in carotid plaque scRNA-seq data. A, UMAP clustering of integrated public carotid plaque scRNA-seq data (10x platform) from Pan et al. and Alsaigh et al. (GSE155512 and GSE159677). **B,** Projection of carotid cells from the mesenchymal supercluster to the UMAP for the coronary data (GSE131778; see Figure 2). Expression of *ACTA2*, *TNFRSF11B*, and *LUM* places cells on an axis of phenotypic diversity from contractile ACTA2+ SMCs to fibroblast-like LUM+ cells, similar to the pattern detected in the coronary atherosclerosis scRNA-seq data. **C,** UMAP clustering of public carotid plaque scRNA-seq data (SmartSEQ2 platform) from Sukhavasi et al. (GSE260657). **D,** Expression of *ACTA2*, *TNFRSF11B*, and *LUM* is consistent with that shown in A and B, but with better detection of *TNFRSF11B* in the intermediate part of the main axis of mesenchymal cell diversity.

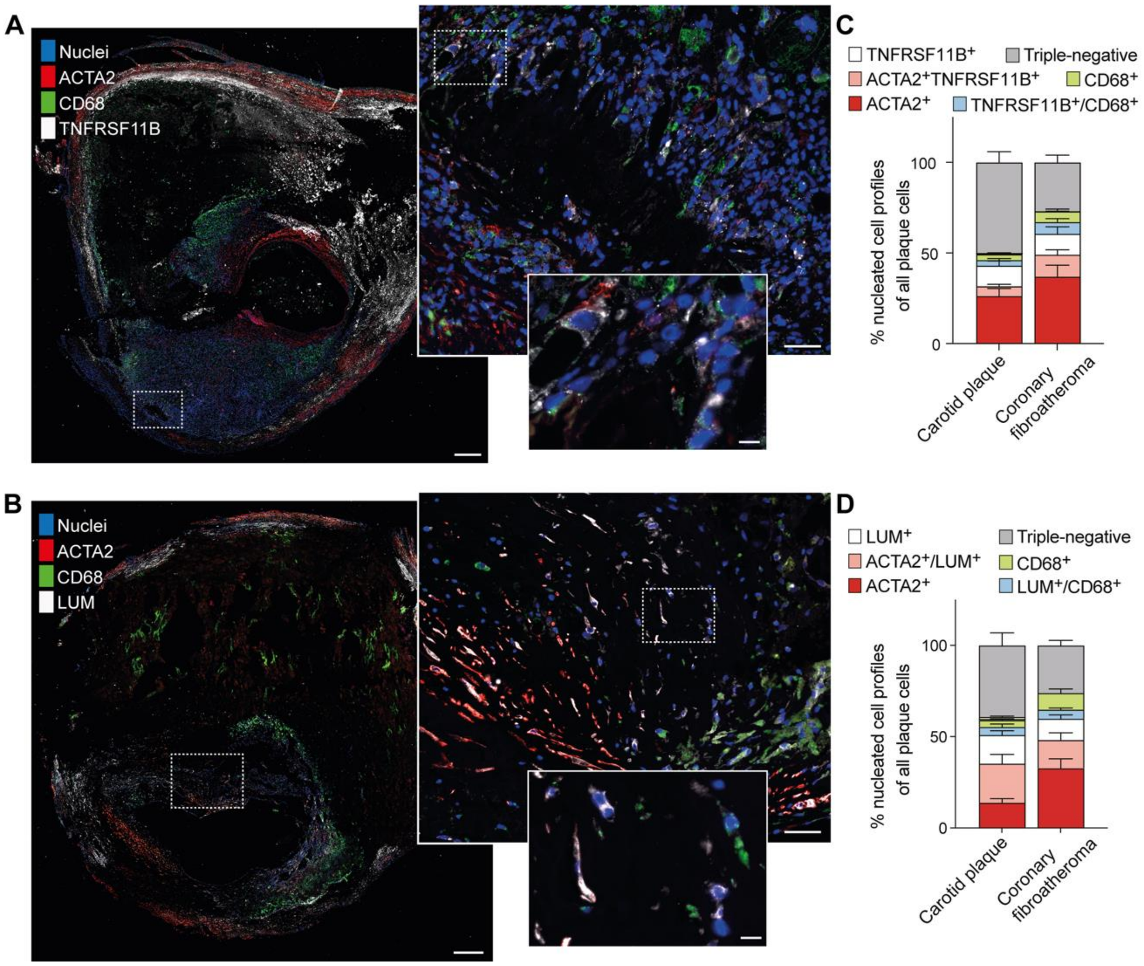

Supplementary Figure 11. Mesenchymal cell types in carotid plaques. A-B, Representative examples of carotid plaque sections stained for ACTA2/CD68/TNFRSF11B and ACTA2/CD68/LUM. The boxed areas are shown at higher magnification. Scale bars, 500 μm, 50 μm, and 10 μm in the low, intermediate, and high-magnification images, respectively. **C-D**, Cell phenotype (marker expression profile) in carotid plaques shown alongside that in coronary fibroatheromas for comparison (n=6 carotid plaques from independent patients analysed, with 1-2 segments per plaque). Bars shown mean and SEM.

Supplementary Table 1. Primary antibodies used in the study.

Target	Clone/ catalogue #	Host	Source	Working dilution
ACTA2	1A4/ M0851	Mouse IgG_{2a}	Dako	1:200
CD45	2B11 + PD7/26 / M0701	Mouse IgG_1	Dako	1:50
CD68	PG-M1/ M0876	Mouse IgG_3	Dako	1:50
LUM	EPR8898(2)/ ab198974	Rabbit monoclonal	Abcam	1:50
OPG	[98A1071]/ NB-100-56505	Mouse IgG_1	Novus Biologicals	1:100
IgG_{2a}	CLCMG2A00	Mouse	Cedarlane lab	conc. matching primary
IgG_1	[B11/6] / ab91353	Mouse	Abcam	conc. matching primary
IgG_3	14474282	Mouse	ThermoFisher	conc. matching primary
IgG	[DA1E]/ 3900S	Rabbit	Cell Signaling Technology	conc. matching primary

Supplementary Table 2. Secondary antibodies used in the study

Target	Clone/ catalogue #	Conjugate	Host	Source	Working dilution
Mouse IgG_3	A21151	Alexa Fluor® 488	Goat	ThermoFisher	1:500
Mouse IgG_3	115-605-209	Alexa Fluor® 647	Goat	Jackson ImmunoResearch	1:500
Mouse IgG_{2a}	A21134	Alexa Fluor® 568	Goat	ThermoFisher	1:500
Mouse IgG_1	A21240	Alexa Fluor® 647	Goat	ThermoFisher	1:500
Mouse IgG_1	A21124	Alexa Fluor® 568	Goat	ThermoFisher	1:500
Rabbit IgG (H+L)	A11011	Alexa Fluor® 568	Goat	ThermoFisher	1:500
Rabbit IgG (H+L)	A21245	Alexa Fluor® 647	Goat	ThermoFisher	1:500
